# CORRIGENDUM (2018; vol 31, no 5, 748-754)

**DOI:** 10.5713/ajas.17.0543C

**Published:** 2021-02-22

The followings are to be changed in Figure 2 and 3 of **Asian-Australas J Anim Sci 2018. Vol 31, No. 5 : 748–754**.

Figure 2 and 3: Experimental time information was added to the top of the figure and in the legend.

**Figure 2:**
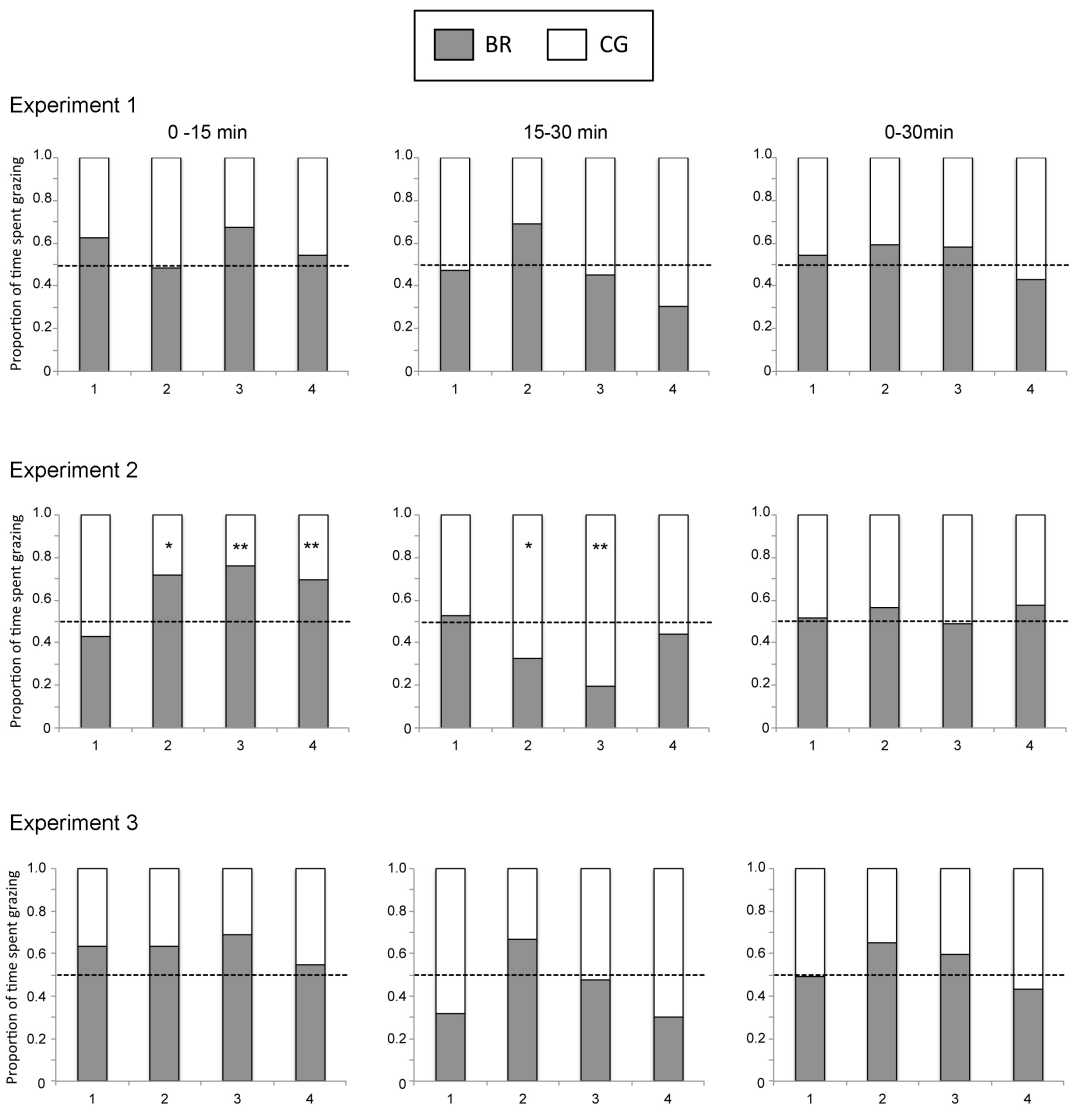
The chronological change for the proportion of time spent grazing ruzigrass tetraploid strain (BR, open columns) hay and Rhodes grass (CG, hatched columns) hay by cattle. The broken lines indicate the proportion of the amount of hay fed with the two grasses (BR:CG = 0.5:0.5). X-axis (1, 2, 3, 4) indicate experimental day. * Indicates a significant bias from the hay fed proportion at p<0.05. BR, *Brachiaria ruziziensis*; CG, *Cloris gayana*.

**Figure 3:**
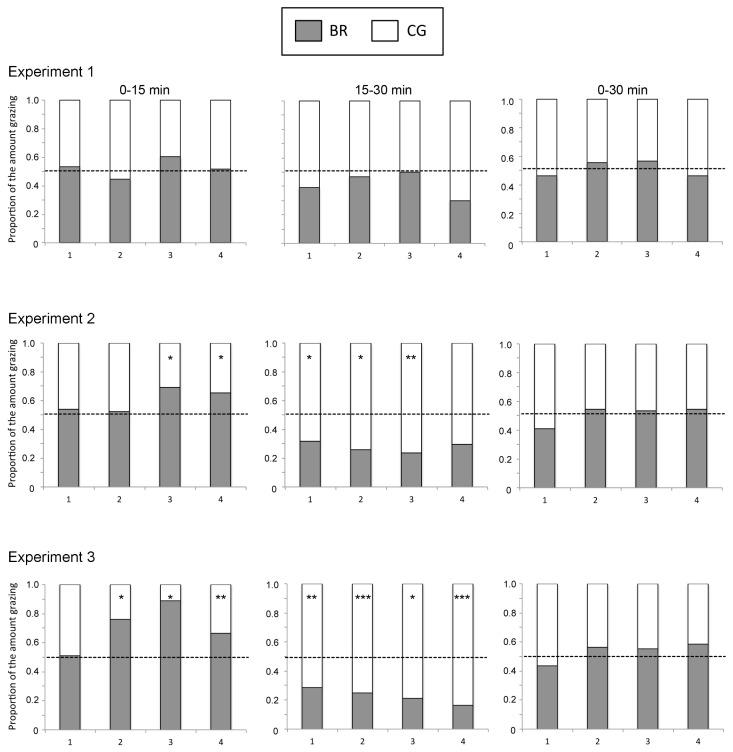
The chronological change for the proportion of grazing amount of ruzigrass tetraploid strain (BR, open columns) hay and Rhodes grass (CG, hatched columns) hay by cattle. The broken lines indicate the proportion of the amount of hay fed with the two grasses (BR:CG = 0.5:0.5). X-axis (1, 2, 3, 4) indicate experimental day. * indicates a significant bias from the hay fed proportion at p<0.05. BR, *Brachiaria ruziziensis*; CG, *Cloris gayana*.

